# Examination of the suitability of collecting in event cognitive processes using Think Aloud protocol in golf

**DOI:** 10.3389/fpsyg.2015.01083

**Published:** 2015-07-28

**Authors:** Amy E. Whitehead, Jamie A. Taylor, Remco C. J. Polman

**Affiliations:** ^1^School of Education, Leisure and Sports Studies, Liverpool John Moores UniversityLiverpool, UK; ^2^School of Psychology, University of Central LancashirePreston, UK; ^3^Centre for Behavioural Change, Psychology Department, Bournemouth UniversityBournemouth, UK; ^4^Institute of Sport, Exercise and Active Living, Victoria UniversityMelbourne, VIC, Australia

**Keywords:** Think Aloud, verbal protocol, motor task, golf, cognitive processes, retrospective recall, verbalizations, methodology in psychological research

## Abstract

Two studies examined the use of Think Aloud (TA) protocol as a means for collecting data of cognitive processes during performance in golf. In Study 1, TA was employed to examine if different verbalisation (Level 2 or Level 3 TA) instructions influence performance of high and low skilled golfers. Participants performed 30 putts using TA at either Levels 2, 3, or no verbalization condition. Although Level 3 verbalization produced a higher volume of verbal data than Level 2, TA at either Level 2 or 3 did not impair putting performance compared to no verbalization. Study 2 examined the congruence between data collected via TA at Level 3 and cued retrospective recall of cognitive processes during golf performance. Experienced golfers performed six holes of golf whilst engaging in Level 3 TA. After performance, three semi-structured retrospective interviews were conducted (10 min after performance, 24 h after performance and 48 h after performance). A comparison of the themes identified large discrepancies between the information reported during TA and at interview, with only 38–41% similarity in variables reported to influence decision making on each hole. Both studies suggest TA is a valuable method for recording cognitive processes of individuals during task performance. TA provides richer verbal data regarding decisions than cued retrospective recall, and TA does not negatively impact performance.

## Introduction

Within the sport psychology literature there is a growing interest in athlete cognitions and how these cognitions underpin behavior (Eccles, 2012). As a result it is important to carefully consider methodologies which are appropriate for collecting this type of data. Ericsson and Simon (1993) introduced the ‘Think Aloud (TA)’ method, which involved asking participants to continuously ‘ TA’ and report their thoughts during the performance of a task. Ericsson and Simon (1993) emphasized the importance of TA in comparison to other methods, such as retrospective recall, due to vital information that may be lost when retrospective reports are used. TA has been used frequently in research to investigate decision making in chess (Gobet and Charness, 2006), medicine (Ericsson, 2004, 2007), nursing (Aitken and Mardegan, 2000), Scrabble (Tuffiash et al., 2007), and algebra tasks (Cook, 2006). However, there is very little research that has used TA for collecting cognitive processes and decision making in sports tasks.

Three types of verbal report protocols have been identified (Ericsson and Simon, 1993). Level 1 verbalization is simply the vocalization of inner speech where the individual does not need to make any effort to communicate his or her thoughts. Level 2 verbalization involves the verbal encoding and vocalization of an internal representation that is not originally in verbal code. For example, verbal encoding and vocalization of scents, visual stimuli, or movement. With this level of verbalization, only the information that is in the participants focus is to be verbalized. Level 3 verbalization requires the individual to explain his or her thoughts, ideas, hypotheses, or motives (Ericsson and Simon, 1993), for example explaining why a certain shot or club is selected in golf.

Within the current sport literature TA has been used to collect data around appraisals and coping in trap shooting (Calmeiro et al., 2010) and golf (Nicholls and Polman, 2008), gender differences in stress, appraisal, and coping in golf putting (Kaiseler et al., 2012) and expert novice differences planning strategies in tennis, (McPherson, 2000; McPherson and Kernodle, 2007). More recently, Calmeiro and Tenenbaum (2011) used TA at Level 2 to investigate differences in the cognitions of experienced and novice golfers during a putting task. Through the use of TA the authors were able to conclude findings such as experienced golfers spent more time than beginners assessing the conditions and planning and experienced players verbalized more diagnostic-related thoughts after the putt and followed these thoughts with planning the next putt. Novice golfers focussed more on the technical aspect of the putt. Whilst this study provides an important insight into skill level differences in cognitive processes of golfers, the very small sample size (3 per condition) limits the generalizability of findings. Calmeiro and Tenenbaum (2011) therefore provided a recommendation that further research needs to identify whether TA interferes with performance.

It has been argued that instructing participants to TA may interfere with thought processes and negatively impact on task performance (Klatzky, 1984), particularly TA at Level 3 (Ericsson and Simon, 1993). A recent meta-analysis by Fox et al. (2011) compared performance on tasks that involved concurrent verbal reporting conditions with their matching silent control conditions. They found that instructing participants to verbalize their thoughts during a task did not alter performance, whereas directing participants to provide explanations for their thoughts actually improved performance. In the meta-analysis by Fox et al. (2011) the majority of tasks were cognitive in nature. To our knowledge no previous studies have examined the influence of TA protocol on motor performance. If TA is to be used more widely to examine cognition in sports it is important to establish if the TA methodology interferes with performance on sport tasks (Calmeiro and Tenenbaum, 2011).

It is also important to consider factors which might potentially moderate the use of TA on motor performance. One such factor is the skill level of the participants. Masters (1992) proposed the theory of reinvestment which suggests that the automisation of a task can be undone or disrupted if a performer tries to control a task or action consciously with declarative knowledge. Fitts and Posner’s (1967) framework of skill learning is part of the reinvestment theory framework. Learning progresses from the declarative, cognitive stage where the performance is cognitively controlled in a step-by-step manner, when learning progresses performance becomes more procedural and automatic which requires little cognitive attention. In the early stages of learning there are rules which the performer cognitively attends to whereas later in the learning process this becomes automatic and the cognitive load changes. Masters (1992) proposed the progression regression hypothesis or reinvestment where high level performance can regress to early stages of skill development in which the execution are more reliant on verbal cues and explicit declarative knowledge (Fitts and Posner, 1967; Anderson, 1982). According to Masters (1992) during progression- regression, a disruption in performance occurs when an ‘integrated’ real time control structure that can run as an uninterrupted (for example, a professional golfers driving off the tee) unit is broken down back into smaller, separate independent units, similar to how it was originally attended to in a step-by-step fashion during the early stages of learning. This in turn slows down performance as each component is run separately instead of all together; as a result there is a gap in each unit which creates more room for error, which would not be present in the integrated autonomous structure (Masters, 1992; Beilock and Carr, 2001). Therefore, it could be argued that by asking a performer who is in the later stages of skill development to verbalize their thought process during performance of a motor skill could result in their skill breakdown. In comparison, Beilock et al. (2002) found that novice or less proficient performers benefitted from attentional monitoring of step-by-step performance.

As a result of the potential limitations previously mentioned to date the majority of research has opted to use retrospective methods to gain insights into thoughts and actions that occur during sport performance. For example, Macquet (2009) studied expert volleyball players and their decision making process using self-confrontational interviews, which were conducted between the 2nd and 5th day following a volleyball match. In addition, Mulligan et al. (2012) used retrospective interviews, prompted by video recordings, to investigate the decision quality in ice-hockey. However, cued retrospective recall of events has a number of important limitations. One issue that has been shown to affect reporting accuracy is memory decay (Ericsson and Simon, 1980; Nicholls and Polman, 2008). In addition, retrospective reports are also distorted by knowledge about success of efforts to resolve stressful events (Brown and Harris, 1978). This can also be linked to the issue of bias as Bahrick et al. (1996) found that recall of student’s high school grades was influenced by the attractiveness of the grade received. Researchers found that the grade A was recalled accurately 89% of the time but the grade D was only recalled 29% of the time.

Tenenbaum and Elran (2003) examined the congruence between actual and retrospective reports for pre- and post-competition emotional states; that were collected 1 h before the event, 30 min after the event and 72 h after the event using a questionnaire. The results revealed that retrospective reports were not affected by the pre–post interference after a 72 h delay. However, athletes underestimated the intensity of post competition unpleasant emotions. In addition, thoughts and feelings that were openly expressed after 72 h were not fully congruent with thoughts and feelings reported in real time. Tenenbaum et al. (2002) also highlighted concerns of whether retrospective reports signify the athlete’s schematic knowledge of how they generally feel before and after a competition. Retrospective measures might be tapping ‘a general schema’ or overlearned set of emotions rather than the particular emotions experienced before an event. Eccles (2012) argues that during retrospective reports participants might be aware of general strategies and recall and report strategies directly and without preference to specific behavior they produced.

The aims of the current studies were mainly methodological in nature. Based on the previous critiques that TA has encountered around interference with performance we first wanted to establish the utility of the TA methodology in the domain of sport in general and golf in particular by examining whether the use of TA at Level 2 or 3 influenced motor performance (golf putting) and the potential moderating effects of skill level. We therefore examined how different levels of TA influence actual performance and information provided by different skill level performers. Hence, based on reinvestment theory differences in performance and verbalisations based on skill level could be expected when using TA in sport.

Secondly, we examined the congruence between TA and the more commonly used retrospective recall methodology.

## Study 1

The first study’s primary aim was to examine whether TA influences motor performance and the potential mediating effects of skill level. Thinking aloud involves continuous reporting of conscious thoughts during the performance of a task. Engaging in TA may promote a self-focus of attention as a performer verbalizes their thoughts and decisions. Masters (1992) suggests that the automatisation of a task can be undone or disrupted if a performer tries to consciously attend to a task. In terms of a motor skill, it can be argued that the level of skill that the performer possesses could also relate to the effect that thinking aloud has on performance. Hence, for skilled performers, which are assumed to be in the automatic phase of skill learning (Fitts and Posner, 1967), it could be suggested that focussing attention on the skill itself degrades performance (Schmidt, 1982) and reinvestment could occur (Masters et al., 1993). It has been proposed that performers in the automatic phase of skill learning will execute the skill in an open-loop fashion where there is little conscious control and processing required. Whereas a novice will be in the closed loop, feedback driven mode, and attention demanding processing (Schmidt and Wrisberg, 2000). Therefore, more experienced golfers may experience more disruption in performance if they engage in TA in comparison to novices due to TA causing the more experienced golfer to attend to their performance in a more step-by-step fashion. However, Fox et al. (2011) found that asking participants to verbalize the task did not impair performance, and when elaborating on the task improvements were found. Study 1 aims to investigate if the use of TA impacts motor performance, by comparing experienced and novice golfers putting performance when using either Level 2 TA, Level 3 TA, or no verbalization. As literature suggests Level 2 verbalization does not have a negative effect on performance (Ericsson and Simon, 1993; Fox et al., 2011), but Level 3 verbalization may affect performance it was predicted that (a) participants in the Level 2 verbalization condition would perform as well as participants in the control condition and (b) that skilled participants in the Level 3 verbalization condition would perform worse than both the control and Level 2 verbalization conditions and novice participants would perform better in the Level 3 verbalization condition than in both the control and Level 2 verbalization conditions. Finally, the content of the verbalizations was analyzed to ensure the TA was carried out according to instructions. Hence, we expected that Level 3 verbalization would result in qualitatively different information as well as quantitatively more information. In addition, the qualitative analysis of the TA data was conducted to provide evidence of possible moderating effects of skill level on performance.

### Methods

#### Participants

Skilled participants were thirty male golfers (age: *M* = 16.9 years, *s* = 0.82; handicap: *M* = 5.3, *s* = 1.51) who attended a further education college in the North of England. Skilled golfers played a minimum of once per week and an average of 8.5 years playing experience. Novice participants were 18 males and 12 females (age: *M* = 21.8 years, *s* = 1.42) who were university students. Novice participants reported they did not play golf on a regular basis and none had played golf in the month prior to testing. Institutional ethical approval was secured and informed consent was obtained from all participants.

#### Apparatus

Novice golfers all used the same right-handed putter, whereas skilled golfers used their own putters. The putting surface was an AstroTurf artificial indoor putting green. The putting hole was a standard size (0.108 m diameter). Thirty of the same brand golf balls were used throughout the testing. A digital voice recorder was used with a small microphone attached to the participant’s collar, and a wire placed inside the shirt connecting to the recording device which was put in the trouser pocket.

#### Procedures

Prior to conducting the experimental procedure all participants took part in a pre-test of putting 10 balls from a 2.50 m distance, which acted to match the participants in the different conditions on ability; by placing an equal ability range of participants into the three conditions (Level 2 verbalization, Level 3 verbalization, no verbalization control) based on the result out of 10 putts. Participants then performed a further 30 putts from a distance of 3 m on an indoor putting green. Instructions for the two TA protocols were adapted to golf putting based upon the guidelines set out by Ericsson and Simon (1993) and Nicholls and Polman (2008). Participants in the Level 2 verbalization group were instructed to say out loud what they were thinking at all times before and after the execution of the putt. Participants in the Level 3 condition were given the same instructions, however, they were also asked to describe and explain their thoughts, providing explanations for their actions. Participants in both conditions were instructed to TA throughout the 30 putts apart from when they were executing the putt. It is important to note that participants were not instructed to verbalize during putt execution to reduce any sort of interference with motor movement (Schmidt and Wrisberg, 2000). If participants were silent for a period of longer than 10 s they would be asked to resume thinking aloud. The third group consisted of a control group and performed 30 putts from the same distance as the other two groups but without any TA instructions. All participants’ scores were recorded, and scores were based on how many putts holed out of 30 putts.

Before the start of the trial, all participants took part in a series of TA exercises to ensure that they could engage in the TA protocol adequately at the level that they were assigned to (Ericsson and Simon, 1993). Both the Levels 2 and 3 verbalization groups completed three tasks: (1) counting the number of dots on a page, (2) an arithmetic exercise, and (3) an anagram problem-solving task. Participants in the Level 2 condition were asked to complete the tasks aloud without explaining how they did them whereas Level 3 participants were asked to also explain how they completed the exercise. Participants took part in these exercises until they had grasped the TA process; which took no longer than 30 min. This exercise took part in meeting room in a close proximity to the putting green.

#### Analysis

##### Performance outcome

A 2 (skill) × 3 (condition) analysis of variance was conducted to explore if there were differences in pre-test putting performance (number of putts holed) between the three groups. To analyze performance on the main putting task the number of putts holed out of 30 was calculated for each participant and a 2 (skill) × 3 (condition) analysis of variance was conducted.

##### Data analysis of content

In order to examine whether Levels 2 and 3 verbalization resulted in qualitatively and quantitatively different information and whether skill level moderated this effect we transcribed verbalizations verbatim. Following checks for relevance and consistency each transcript was subjected to a line by line content analysis by the first author to identify statements which related to the cognitive process of each shot played. Individual elements of ‘meaningful information’ were considered and coded. Similar to Nicholls and Polman (2008) the verbalizations by the participants that were coded were relevant to the task, which in this case meant verbalizations associated with golf performance. Data which were not relevant to the task, such as verbalizations about what an individual had eaten last night, a loved one, and their favorite football team, were removed from the data set. Units of information were coded according to categories derived from a modified version of Calmeiro and Tenenbaum’s (2011) coding scheme (see **Table 1**). This coding scheme was used and adapted as it was one of the only studies to have examined cognition processes using TA in golf putting previous to this paper. The second author independently analyzed a 10% sample of verbal data; the inter-rater agreement was 89%.

**Table 1:** Coding scheme framework adapted from Calmeiro and Tenenbaum (2011).

Theme	Description	Example of raw data quote
Gathering information	Reflected participants’ search for relevant characteristics of the environment	“There’s a break left,” “there’s a ridge on the middle of the green”
Planning	Reference to planning a shot, for example targets to aim for, power of putt.	“Need to aim more right,” “I need to be a bit more firm”
Technical instruction	Specified technical aspects of the performance	“Arms bent,” “feet are parallel”
Reflection	Reflected on what had happened in terms of process or evaluation of the putt	“Just missed left,” “it broke at the end,” “yes, good putt”
Self-encouragement	Refers to any positive words relating to self-encouragement.	“You can do this,” “concentrate on this”

A 2 (skill) × 2 (condition) ANOVA was conducted to explore the difference in the amount of data (words) produced during Levels 2 and 3 verbalization and the two ability levels. Based on the an adaptation of Calmeiro and Tenenbaum (2011) coding scheme the units of information that were coded were analyzed using a 2 (skill) × 2 (condition) MANOVA to investigate the difference in the total frequency of themes that were verbalized during Levels 2 and 3 verbalization for both experienced and novice golfers. Significant multivariate effects were followed up with univariate ANOVA and independent *t*-tests with Bonferroni correction.

### Results

#### Performance

The first 2 (skill) × 3 (condition) ANOVA examined pre-test performance (number of successful puts out of 10 attempts). As expected there was a significant main effect for skill [*F*(1,54) = 10.73, *p* = 0.002, $\eta_{p}^{2}$ = 0.17] with skilled players (*M* = 4.83,SD = 2.23) outperforming novice players (*M* = 3.03, SD = 1.87), however, there was no significant main effect for condition [*F*(2,54) = 0.05, *p* = 0.953, $\eta_{p}^{2}$ = 0.002] or interaction between skill and condition [*F*(2,54) = 0.01, *p* = 0.989, $\eta_{p}^{2}$ < 0.001]. This finding implies that pre-test performance across conditions was equivalent.

The second 2 (skill) × 3 (condition) ANOVA analyzed test performance (number of successful putts out of 30 attempts). For descriptive statistics see **Table 2**. A significant main effect was found for skill [*F*(1,54) = 20.76, *p* < 0.001, $\eta_{p}^{2}$ = 0.28]. Skilled golfers performed better (*M* = 10.97, SD = 4.82) than novice golfers (*M* = 5.87, SD = 3.96). No significant main effect was found for condition [*F*(2,54) = 2.79, *p* = 0.08, $\eta_{p}^{2}$ = 0.09] and there was no significant interaction between skill and condition [*F*(2,54) = 0.28, *p* = 0.75, $\eta_{p}^{2}$ = 0.01].

**Table 2:** Mean and (SD) test performance (successful putts out of 30) as a function of skill and condition.

Skill	Control	Level 2	Level 3
Skilled	9.40 (3.24)	12.30 (4.97)	11.20 (5.92)
Novice	3.70 (3.65)	6.60 (3.89)	7.30 (3.74)
Total	6.55 (4.45)	9.45 (5.24)	9.25 (5.22)

#### Verbalization Content

**Table 3** presents descriptive statistics for the volume of verbal data provided (number of words) during test performance for the skilled and novice golfers in the Levels 2 and 3 verbalization conditions. A 2 (skill) by 2 (condition) ANOVA showed a significant condition main effect [*F*(1,36) = 66.31, *p* < 0.001, $\eta_{p}^{2}$ = 0.64]. Level 3 verbal protocol resulted in significantly more words verbalized (*M* = 385, SD = 110) compared to Level 2 verbal protocol (*M* = 141, SD = 69). There was no significant main effect for skill [*F*(1,36) = 0.01, *p* = 0.89, $\eta_{p}^{2}$ < 0.001] nor was there a significant interaction [*F*(1,36) = 0.03; *p* = 0.85, $\eta_{p}^{2}$ = 0.001].

**Table 3:** Mean and (SD) volume of verbal data provided (number of words) during test performance as a function of skill and condition.

Skill	Level 2	Level 3
Skilled	136.70 (73.99)	385.30 (122.58)
Novice	145.90 (67.05)	383.80 (103.42)
Total	141.30 (68.88)	384.55 (110.38)

Once verbal data was thematically analyzed, the frequency of verbalization of each theme was compared with a 2 (skill) × 2 (condition) MANOVA. **Table 4** shows descriptive statistics for the frequency of verbalization of each data theme for the skilled and novice golfers in the Levels 2 and 3 verbalization conditions.

**Table 4:** Mean and (SD) frequency of verbalization of each data theme during test performance as a function of skill and condition.

Measure	Level 2		Level 3	
	Skilled	Novice	Skilled	Novice
Gathering information	1.00 (1.33)	0.00 (0.00)	3.00 (2.00)	0.70 (1.64)
Self-encouragement	2.30 (2.36)	4.40 (3.81)	3.10 (3.03)	2.40 (1.86)
Planning	10.30 (7.46)	7.00 (4.00)	15.30 (3.43)	11.00 (6.56)
Reflection	10.20 (3.73)	15.40 (7.47)	16.10 (6.34)	16.80 (5.12)
Technical instruction	4.40 (4.79)	1.60 (1.89)	2.80 (2.89)	8.90 (3.14)

There was a significant multivariate interaction between skill and condition [Wilks’ λ = 0.46, *F*(5,32) = 5.15, *p* = 0.001, $\eta_{p}^{2}$ = 0.46], with univariate ANOVA’s indicating an interaction only for the theme Technical Instruction [*F*(1,36) = 17.68, *p* < 0.001, $\eta_{p}^{2}$ = 0.33]. Independent t-tests with Bonferroni correction indicated novice golfers verbalized more about technical instructions than skilled golfers in the Level 3 condition [*t*(18) = 4.51, *p* < 0.001], but not in the Level 2 condition [*t*(18) = 1.72, *p* = 0.103].

There was a significant multivariate effect for skill [Wilks’ λ = 0.65, *F*(5,32) = 3.41, *p* = 0.014, $\eta_{p}^{2}$ = 0.35] with skilled golfers verbalizing more frequently than novice golfers about gathering information [*F*(1,36) = 12.24, *p* = 0.001, $\eta_{p}^{2}$ = 0.25] and planning shots [*F*(1,36) = 4.56, *p* = 0.04, $\eta_{p}^{2}$ = 0.11].

There was a significant multivariate effect for condition [Wilks’ λ = 0.40, *F*(5,32) = 9.56, *p* < 0.001, $\eta_{p}^{2}$ = 0.60], with more verbalization in the Level 3 than Level 2 condition about gathering information [*F*(1,36) = 8.19, *p* = 0.007, $\eta_{p}^{2}$ = 0.19], planning shots [*F*(1,36) = 6.39, *p* = 0.016, $\eta_{p}^{2}$ = 0.15], and technical instruction [*F*(1,36) = 2.43, *p* = 0.011, $\eta_{p}^{2}$ = 0.17].

### Discussion

The results of Study 1 showed contrary to predictions that the use of verbalization at either Level 2 or Level 3 did not significantly influence performance across skill level. This indicates that Level 3 TA verbalization, requiring explanations of a performers thought processes, is not associated with decreases in motor performance in comparison to Level 2 TA verbalization or no-verbalization irrespective of the skill level of the performer. Examination of mean performance actually suggested performance was slightly better in the TA conditions than control. From a theoretical perspective, reinvestment theory (Masters, 1992) suggests that, TA has the potential to negatively influence performance among skilled performers. By instructing golfers to TA their attention could be directed to the step-by-step mechanics of the skill which has been associated with poorer performance. In the present study there was no clear evidence that thinking aloud resulted in skilled performers reinvesting in explicit rules. Skilled performers verbalized few technical instructions in either the Level 2 or Level 3 TA conditions, with the majority of verbalizations focused on planning and evaluating shots. This finding suggests TA does not result in reinvestment and does not lead to performance breakdown. However, a possible explanation for the converse performance effect found in the higher skilled group is that the golfers did not dwell on mistakes or technical errors (reminisce/ruminate) but that TA helps them to actively seek solutions as indexed by greater use of deliberate planning and gathering information. Because of their greater knowledge base, skilled golfers are more likely to use the information and planning to their advantage and enhance their performance (see Gagné and Smith, 1962 for similar results). The absence of reporting of increased technical information during TA at Level 3 in the skilled group might also explain the lack of performance decrements in this group. Hence, reinvestment theory suggests that it is particularly the monitoring or the actual execution of the motor skill which might result in skill breakdown and performance decrements (Masters, 1992).

Further analysis of the content of the data revealed that Level 3 verbalization produced a larger amount of verbal data than Level 2 independent of skill level. In addition, differences were apparent between Levels 2 and 3 TA and between skilled and novice participants. This suggests that Levels 2 and 3 TA results in qualitatively and quantitatively different information and skill level also influences the information provided. In particular, skilled golfers verbalized more about gathering information and planning before taking a putt than novices. Whereas novices verbalized more about the technical aspects of their performance compared to skilled golfers, but only when using TA at Level 3. Similar results were reported by Calmeiro and Tenenbaum (2011) where beginners focussed on mainly technical aspects during TA and high level golfers reported greater use of gathering information and planning. Similarly, Beilock et al. (2002) found that experts made less reference to putting mechanics in their episodic recollections than novices. Beilock et al. (2002) proposed that a novice’s performance of a skill is based on declarative knowledge that is held in working memory and is attended to in a step-by-step fashion (Fitts and Posner, 1967; Anderson, 1983, 1987). Low level golfers are likely to be in the cognitive phase of learning (Fitts and Posner, 1967) which is characterized by using explicit, technical information to guide skill execution whereas the high level golfers are more likely in the autonomous phase of skill learning. This stage is characterized by the use of implicit knowledge. Therefore, by examining the content on the verbalisations clear differences have been identified between the level of TA used and the expertise of the performer.

The results of Study 1 showed that, independent of skill level, performance was slightly better in the TA conditions in comparison to the control condition. A possible explanation for this observation is that TA resulted in the participants spending more time planning and evaluating their performance. This in turn might have resulted in developing strategies which enhanced performance. Future studies might examine additional factors which might result in difference between TA and control, including eye-movement or behavioral aspects.

To our knowledge this is the first study which has demonstrated that TA at Level 3 does not impede motor performance (Calmeiro and Tenenbaum, 2011) and results in qualitatively and quantitatively different information than that obtained by Level 2 TA. The information provided at Level 3 appears to be richer in detail and skill level moderates the information obtained at both Levels 2 and 3 TA. However, study one cannot establish whether TA is a more appropriate methodology of collecting data on cognitive processes during sporting performance compared to other methodologies like retrospective recall. We therefore conducted a second study the further examine the utility of TA methodology.

## Study 2

Study 2 was designed to compare the TA method with the more commonly used retrospective recall. A lot of decision making and cognitive processing research has adopted a retrospective method (Macquet, 2009; Cotterill et al., 2010; Mulligan et al., 2012). However, if data can be collected during performance of a task it is thought this will minimize the event-recall period and increase the likelihood of collecting accurate data (Folkman and Moskowitz, 2004). This study aimed to examine the congruence between the verbal data generated by golfers when using TA and that generated by retrospective interviews. Participants performed six holes of golf whilst engaging in Level 3 TA. After performance, three semi-structured retrospective interviews were conducted, 10 min after performance, 24 h after performance, and 48 h after performance. It was predicted that with increasing time there would be less correspondence between verbal data captured using TA and retrospective recall. No prediction was made with regard to the volume of information provided.

### Methods

#### Participants

Participants were six male golfers (M age 30.5 years, M handicap 5.5, M playing years 19), and all members of the same golf club. The participants were recruited via a sign-up sheet in the club house and all participants were volunteers. Institutional ethical approval was secured and informed consent was obtained from all participants.

#### Materials

Each golfer played with their own golf clubs on the same six holes of the same golf course. As in Study 1, participant’s verbalizations were recorded using a Sennheiser USA ENG G3 wireless digital voice recorder. A score card was used to mark the number of shots taken on each hole.

#### Procedure

Participants were asked to meet 1 h prior to golf performance in order to be briefed and to take part in a series of TA exercises (see Study 1). Each of the golfers then played six holes of golf accompanied by a researcher. During all six holes, participants were asked to describe their thoughts before and after the execution of each shot and provide an explanation for their actions (Level 3 TA). Golfers were told that they could engage in TA between holes if they had any thoughts they wished to verbalize. Participants were instructed to, “Think Aloud and say everything that comes into your head before and after each shot you take. Every time you TA can you please explain this thought.” If they were silent for a period of longer than 20 s they were asked to resume TA. The thoughts were recorded until the golfers had completed all six holes. Each golfer played on the same golf course with their own golf clubs, although the six holes played were varied for each golfer.

Following the completion of the six holes each participant was then asked to take part in three semi-structured interviews, the first being approximately 10 min after performance of the six holes. The second interview was held 24 h and the third 48 h after performance. Each interview involved asking semi-structured questions about the decision making that occurred during two separate holes for each interview. The selection of the holes for retrospective recall were conducted in a random manner. It is believe that random selection provides a far better way of doing this than selecting holes on a number of criteria of which we do not know if they have an influence or not on what is recalled. Although there is some suggestion that success might be better remembered, currently there is little evidence that there are differences in remembering events which are more or less successful in sport. For consistency the same sets of questions were asked to each participant, however, participants were free to answer in any way they wanted. Both undirected and directed questions were used during interviews (Eccles, 2012). Questions asked during these semi structured interviews were, “please can you describe hole…,” “can you tell me what you were thinking about during your first shot?,” “What kind of shot did you play and why?,” “what club did you use and why?,” “were there any environmental factors that affected your shot?” Each interview was conducted at the same time of day and took approximately 20 min to complete. It is important to note that undirected questions such as “can you tell me what you were thinking during your first shot?” were used to try and provide information of higher validity because the question avoids constraining the participant to interpret thoughts (Eccles, 2012).

#### Data Analysis

Each participant’s verbal reports from TA and interviews were transcribed verbatim. Following checks for relevance and consistency each transcript was subjected to a line by line content analysis by the first author to identify statements which related to the decision making process of each shot played. Individual elements of ‘meaningful information’ were considered and coded. Similar to Nicholls and Polman (2008) the verbalizations by the participants that were coded were relevant to the task, which in this case meant verbalizations associated with golf performance. Data which were not relevant to the task, such as verbalizations about what a participant had eaten the previous night, a loved one, and their favorite football team, were removed from the data set. Units of information were coded according to categories derived from a modified version of Nicholls and Polman’s (2008) coding scheme (see **Table 5**). Thirty-four first-order themes were initially identified and then related themes were grouped into 11 second-order themes. A more detailed coding scheme was used in Study 2 with a large number of first-order themes to represent the wide range of variables considered by participants when making decisions on a real golf course. The second author independently analyzed a 10% sample of the raw data using the coding scheme developed by the first author. The Level of agreement between the first and second author was 71%. Any discrepancies were discussed and an agreement was reached.

**Table 5:** First and second-order themes identified from Think Aloud (TA) and interview data.

Second-order theme	First-order theme	Description	Example of ‘raw’ data quote
Course conditions	Quality of greens	Mention of grass length, or obstacles on the green which could affect the run of the ball	“The green has been sanded so it’s bobbly”
	Course hazards	Anything stopping the player’s view the green or anything which could disturb play	“Can’t see the hole because of the huge mound in front of me”
	Rough	Being in the rough	“I finished on the left side of the rough”
Course management	Lie of ball	When the golfer refers to the lie of the ball	“the lie is not the best”
	Playing bunker shot	Being in the bunker	“It’s in the bunker that’s horrendous”
	Club selection	Any reference to which club has been selected	“I’m using a driver because…”
	Pin position	Where the pin is located on the green	“It’s a blue flag which indicates the pin is at the back portion of the green”
	Movement of Green	How the ball will move on the green	“It’s going to move left to right”
	Distance to pin Tee position	How far the shot being played is from the pin Where the tee is positioned on the green	“This is a 350 yard drive to the green” “the tee is toward the back of the green”
Distractions	Having to wait at tees	Waiting to play a shot due to either slow play or green keepers	“I could do with that old fella hurrying up”
	Researcher	Referring to the researcher	“I feel an idiot doing this in front of you”
	Dirt on the ball	Having any form of mud or dirt on the ball	“The ball was dirty”
	Temperature	Any reference to how hot or cold the player is	“I’m really hot under this hat”
Environment	Wind	Wind is considered in relation to shot decision.	“The wind is moving left to right and slightly into so I am going to use….”
	Tree	A tree obstructing the intended line of next shot or is taken into consideration of next shot.	“The trees are reachable from this tee”
	Rain	Any reference to rain	“It’s starting to rain but it’s not too bad”
Mistakes	Shot error	Any reference to a shot error after shot has been taken. Either physical (swing fault) or mental (club selection)	“I’ve hit that too hard”, “I’ve fluffed that” “I should of used a … iron”
Performance	Result of shot Happy with the shot	Describing how the shot has finished Positive statements about the shot just played	“That’s finished up on the fairway” “that’s exactly how I wanted to hit it”
	Negative words	Using any kind of negative words or cursing before or after a shot has been taken	“I am a rubbish golfer”
	Short putts	Putts from within 5 feet	“It was about three foot and I just stroked it in for a par”
Score	Score	Any reference to score for the hole or round	“I needed to putt this for a birdie”
	Number of putts	Concerns about the number of putts played during the hole or round.	“I’m going to try and 2 putt par”
Safety	Play safe	Choosing to play a safer or less cautious shot.	“I need to play a safe shot here”
	Risk	Playing a shot with a more high risk element.	“There’s a bit of risk in this shot but if it pays off it will be worth it”
Pre-performance	Practice swing	Taking a practice swing before hitting the shot	“A couple of practice strokes looking at the hole”
	Cleaning the ball	Cleaning the ball before the next shot	“I will just clean the ball up”
	Pre-performance words	Words said before shots are played	“OK ready”
	Targets to aim for Overall aim of shot	Objects or parts of the course that are used as targets for shots. Specifying exactly what is intended in the shot	“I’ve picked the church steeple in the background to aim for” “I want this to bend round the tree and then I can chip it onto the green”
Reflection	‘Last time I played this shot’	Any reference to what they did previously (last shot or last week) when playing a similar shot	“Like the last putt, I don’t have to worry about the pace too much”
	‘Last time I played this hole’	Any reference to what they did previously when playing the same hole.	“Last time I played this hole, I hit it onto the road”
Feelings/Emotions	Anxious	When a golfer refers to being nervous or anxious	“I’m always a bit anxious on the first shot of the first hole.
	Confidence	Stating that the performer is feeling confident about a shot or hole	“I know I can hit this shot well”

##### Number of themes

The number of first-order themes each participant identified as influencing their decision making on each hole was calculated for the TA and interview data. A 2 (condition) × 3 (time) repeated measure ANOVA was conducted to identify any differences between the number of themes identified via TA and interview. A significant interaction was followed up by paired samples *t*-tests to compare TA and interview at each time point. The alpha level was set at *p* < 0.05.

To establish how the data collected at TA and interview differed, the most frequently cited second-order themes using each method of data collection were identified. Frequency of citation of each theme between TA and interview were calculated for each interview time point. Multiple 2 (condition) × 3 (time) repeated measures ANOVA’s were conducted to investigate how the second-order themes identified at TA and interview differed at each time point (10 min post performance, 24 h post and 48 h).

##### Similarity of themes

Percentage similarity of first-order themes identified on each shot during TA and interview was calculated at each interview time point. A one-way repeated measures ANOVA was then conducted to determine any significant differences in the percentage similarity between TA and interview across the three interview time points.

### Results

#### Number of Themes

**Table 6** displays the mean number of first-order themes that golfers identified as influencing their decision making during two holes of golf, with a comparison made between data collected using TA and interview at the three interview time points. A 2 (condition) × 3 (time) repeated measures ANOVA indicated that there was a significant interaction between condition and time [*F*(2,10) = 9.49, *p* = 0.005, η^2^ = 0.65]. Follow up paired samples t-tests indicated that quantitatively the mean number of first-order themes identified during TA did not differ from the number identified during the interview 10 min after performance or 24 h after performance (*p* > 0.05). However, significantly more themes were identified via TA than at interview 48 h after performance [*t*(5) = 3.44; *p* = 0.018, *d* = 0.43].

**Table 6:** Number of first-order themes identified during TA of two separate holes (during performance) and interview at the three time conditions; 10 min, 24h, and 48 h after performance.

Think Aloud over two separate holes			Interview	
*M*	SD	Time of Interview	*M*	SD
37.50	8.73	10 min	35.33	16.51
41.00	8.27	24 h	36.00	11.61
51.21	16.31	48 h	37.21	9.45

**FIGURE 1:**
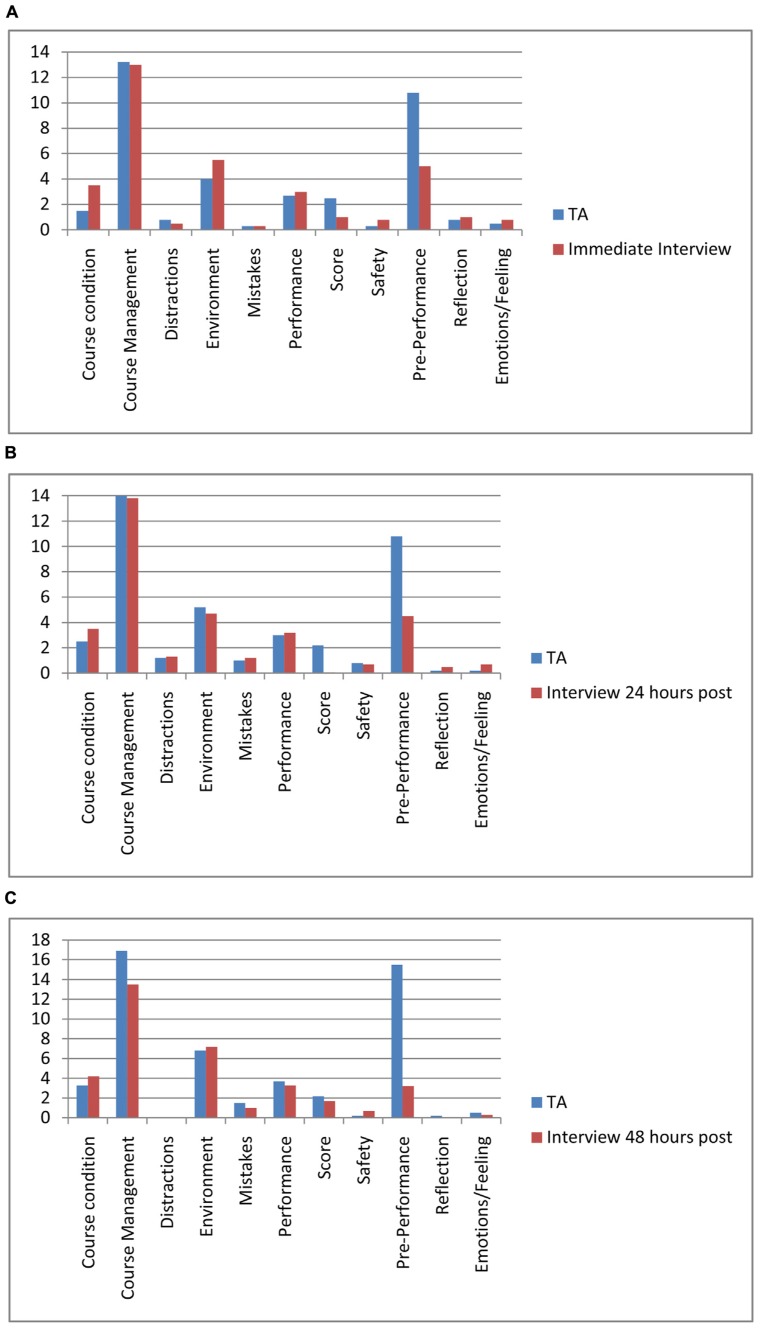
**Mean frequency of second-order themes that were cited during Think Aloud (TA; during performance) and interviews; **(A)** 10 min post performance **(B)** 24 h post-performance **(C)** 48 h post performance**.

**Figure 1** shows the difference between TA and interview in the numbers of times each second-order theme was identified as a variable influencing a decision, with comparisons displayed for interview 10 min post performance vs. TA (1a), 24 h post performance interview vs. TA (1b), and 48 h post performance interview vs. TA (1c). A 2 × 3 repeated measures ANOVA was conducted for each of the 11 second-order themes to investigate how the second-order themes identified at TA and interview differed at each time point. There was a condition main effect for the second-order theme Score [*F*(1,5) = 15.92; *p* = 0.01, η^2^ = 0.76] in that more verbalizations were made about the score during TA than interview. In addition there was a condition main effect for the second-order theme Pre-performance [*F*(1,5) = 39.71, *p* = < 0.001, η^2^ = 0.88], with more verbalisations made about Pre-performance activity during TA than during interview. Finally, there was a significant interaction between time and condition for the variable pre-performance [*F*(2,10) = 6.31, *p* = 0.02, η^2^ = 0.56]. Follow up paired samples *t*-tests found that pre-performance activity was cited more frequently in TA reports than at interview 10 min after performance [*t*(5) = 9.71, *p* < 0.001, *d* = 1.60], 24 h after performance [*t*(5) = 3.00, *p* = 0.030, *d* = 1.61] and 48 h after performance [*t*(5) = 5.89, *p* = 0.002, *d* = 3.55]. The largest difference was between TA and the interview 48 h after performance as indicated by the larger effect size. All other main effects and interactions were non-significant (*p* > 0.05).

#### Similarity of Themes

**Table 7** shows the percentage similarity between TA and interview for the thoughts (first-order themes) verbalized by golfers during decision making on each shot played. Percentage similarity at each time point was relatively low (ranging between 38 and 41%), suggesting participants reported different thoughts during TA than at interview. One-way repeated measures ANOVA indicated no significant difference in percentage similarity across the three time points [*F*(2,17) = 0.09; *p* = 0.91, η^2^ = 0.01].

**Table 7:** Percentage similarity in themes identified during TA and at interview.

	Percentage of similarity	
Comparison	*M*	SD
TA vs. 10 min interview	40.66	8.73
TA vs. 24 h interview	37.83	14.83
TA vs. 48 h interview	40.16	12.04

### Discussion

The purpose of Study 2 was to test the methodology of TA further by examining the congruence between verbal data collected on decision making in golf via Level 3 TA reports and cued retrospective recall at different time intervals after performance. Results found similarities in the quantity of data provided, however, the congruence between thoughts verbalized during TA and interview was low, with only a 38–41% level of agreement. This suggests participant’s verbalized different thoughts during TA than they did during retrospective interview. The level of congruence between TA and retrospective interview remained low whether interviews were conducted 10 min after performance, 24 or 48 h and did not change as a function of time of interview as predicted.

When thoughts were recorded using TA golfers talked more about their score and the pre-performance activity they engaged in prior to a shot compared to at interview. This included thoughts about a practice swing, cleaning of the ball, pre-performance words or identifying a target to aim for. These verbalizations are important for understanding the decisions golfers make during a round and were missed when data was collected at interview.

The results of Study 2 indicate a clear discrepancy in the verbalizations made by golfers regarding their thoughts and decisions when verbal data is collected using TA compared to retrospective interview. This suggests retrospective interviews that have been previously used (Macquet, 2009; Cotterill et al., 2010; Mulligan et al., 2012) may be limited for understanding cognitions involved in decision making in sport.

## General Discussion

The findings from both Study 1 and 2 support the use for TA as an appropriate method for collecting in event cognitions. Recent research (Calmeiro and Tenenbaum, 2011) has expressed a need to determine whether verbalizing during motor performance (putting) interferes with task outcome. This was examined in Study 1. In this study the moderating effects of skill level was also considered. Hence, based on Masters (1992) theory of reinvestment it was predicted that those in the higher skilled stages of learning would experience a larger performance decrement due to the possibility that what would usually be an automatic task could be disrupted or undone if the performer tried to control the task with declarative knowledge using TA. The results of Study 1 demonstrated that contrary to previous findings (Klatzky, 1984) and Masters (1992) reinvestment theory, thinking aloud at Level 3 does not have a negative effect on motor performance. Hence, it was expected that relatively novice performers would benefit from thinking aloud at Level 3 due to the increased attentional monitoring of their step-by-step performance (Beilock et al., 2002) whereas skilled performers would show a decrement in performance due to experiencing a disruption in their automatic processes of the putting skill (Masters, 1992). The latter prediction was based on the notion that skilled performance is said to be controlled in an open loop fashion and use of explicit knowledge would interfere with performance (e.g., Masters et al., 1993). For novice performers on the other hand, performance is more likely characterized as closed loop and feedback driven, requiring attention demanding processing. Hence, hypothesis testing is an important aspect of skill acquisition during the early stage of the learning process (Fitts and Posner, 1967). However, Level 3 TA did not disrupt performance in either skill level group and did not lead to reinvestment among skilled performers since there were very few verbalizations regarding technical instructions. It is thought that the higher skilled performers may have been using deliberate planning and gathering information to enhance their performance.

Study 2 provided further evidence for use of TA as a methodology within the sport research domain and that retrospective interviews are potentially limited for understanding cognitions related to decision making in sport since large discrepancies were found between thoughts generated during TA and during retrospective interviews. Only a 38–41% similarity was found in thoughts verbalized by golfers during TA and at interview. These findings could be accounted for through memory decay at interview. Research by Stone et al. (1998) has found that 30% of participants failed to retrospectively report things that they had reported during in event assessments. Further explanations could be the issue of bias at interview, since participants might report shots differently depending on their perceived success. Bahrick et al. (1996) found issues of bias in terms of student’s high school grades and those who achieved higher grades were more accurate in recalling them. This study also takes a step forward in terms of ecological validity as it involves using TA in a real life golf setting, where golfers are performing on a real course using all range of shots, which is a factor that limits a lot of TA literature (Calmeiro and Tenenbaum, 2011).

The present studies provide the first evidence that using the TA methodology does not negatively influence motor performance of a self-paced task and that TA appears to be a more suitable methodology than retrospective recall when examining cognitions in the domain of sport.

Previous research using TA (Nicholls and Polman, 2008; Calmeiro and Tenenbaum, 2011; Germain and Tenenbaum, 2011) have treated TA as a record of the participant’s ongoing decision making process, and they believe that the information verbalized represents a portion of the information currently being attended to (Ericsson and Simon, 1980). However, it is important to acknowledge that we cannot be 100% certain that everything being verbalized is what is actually being thought at the time. Furthermore, some cognitive processes are not conscious and an individual cannot access what happens to the decision making process outside of awareness. Unconscious processes are not possible to verbalize and the mechanisms that mediate the process of unconscious processing are still widely discussed in cognitive psychology (Nisbett and Wilson, 1977; Dehaene et al., 1998; Elsner et al., 2008). For example, Nisbett and Wilson (1977) highlighted how an individual’s ability to provide valid reports of cognitive processes could prove difficult. Nisbett and Wilson (1977) argue that there are limits to what can be accessed consciously. Instead participants may give implicit theories about their thought process. If a performer does not have direct verbal access to their control process, especially if they are automatic, it may lead to a performer reporting cues that they expect or have been told are important. As such, golfers might report what they think they should do rather what they actually do. Problems associated with Level 2 TA is that there is no independent means of assessing their completeness (Wilson, 1994) as under some circumstances because some cognitive processes are not part of focused attention, or appear in a form that is not easily verbalizable (Ericsson and Simon, 1993). Therefore, Level 2 TA verbalisation may not provide enough detail of the thought processes involved in decision making during golf performance.

Ball et al. (2015) highlighted the disruptive effect that can occur through verbally reporting as verbal-overshadowing occurs as the formation of a verbally biased memory representation that overshadows original visual memory. It is argued that by asking participants to explain and describe their thoughts, as is required in Level 3 verbalization, this may result in reactive effects on task process that can influence the performance of a task. However, Ball et al. (2015) research focused on an insight problem solving task, whereas golf involves more simplistic reasoning regarding shot selection. As such the nature of a task should be considered in future research that examines the influence of TA on task performance.

## Conclusion

Collective findings suggest TA does not influence motor performance and is a valuable method for recording cognitive processes of individuals during task performance. Findings revealed that Level 2 TA is different from Level 3 TA with Level 3 providing richer information in both the quantity and quality of the information. However, this appears to be moderated by the skill level of participants. Furthermore, TA provides richer verbal data regarding decisions than cued retrospective recall. Future research should use TA from a theoretical perspective to examine in-event cognition in self-paced sporting tasks such as golf. Future studies might examine skill level differences in cognition for golfers playing a full round of golf on a real course, and examine the influence of competitive stress on cognition. This would advance understanding of in event cognition in sport, since much of the research examining cognitive processes in sport is conducted in contrived laboratory environments or using retrospective methods.

## Conflict of Interest Statement

The authors declare that the research was conducted in the absence of any commercial or financial relationships that could be construed as a potential conflict of interest.
